# Integrated Management of Energy, Wellbeing and Health in the Next Generation of Smart Homes

**DOI:** 10.3390/s19030481

**Published:** 2019-01-24

**Authors:** Saad EL Jaouhari, Emilio Jose Palacios-Garcia, Amjad Anvari-Moghaddam, Ahmed Bouabdallah

**Affiliations:** 1 IMT Atlantique, IRISA, UBL, F-35576 Cesson Sévigné, France; saad.eljaouhari@imt-atlantique.fr (S.E.J.); ahmed.bouabdallah@imt-atlantique.fr (A.B.); 2 Department of Energy Technology, Aalborg University, DK-9220 Aalborg East, Denmark; epg@et.aau.dk

**Keywords:** smart home, healthcare, wellbeing, energy management, Internet of Things, security and privacy

## Abstract

This contribution proposes an implementation for next generation smart homes, where heterogeneous data, coming from multiple sensors (medical, wellbeing, energy, contextual, etc.) and house equipment (smart fridge, smart TV, etc.), need to be managed, secured and visualized. As a first step, it focuses only on energy and health data. However, it aims to lay the foundations to manage any type of information towards the development of smart interactions with the house, which might include artificial intelligence and machine learning. These data are securely collected using a central Web of Things gateway, located inside the smart home. For the e-health part, a set of possible use-cases is provided, along with the current progress of the implantation. In this regard, the main idea is to link the next generation smart homes with external medical entities in order to provide, first, quick intervention in the event of an abnormality being detected, and to be able to provide basic medical services such as remote consultations with a doctor for a particular health issue. This vision can be very promising, particularly in rural areas, where access to medical services is difficult. As for the energy part, the aim is to collect users’ energy consumption inside the smart home, which can be supplied from different sources (heat, water, gas, or electricity), and to enable the use of advanced algorithms to predict and manage local energy consumption and production (if any). This approach combines data collected from smart meters, operational information of the smart energy devices (the status of smart plugs), user’s requests and external network signals such as energy prices. By using a home energy management system that accepts such input parameters, the operation of in-home devices and appliances can be optimally controlled according to different objectives (e.g., minimizing energy costs and maximizing user’s comfort level).

## 1. Introduction

The concept of smart homes has undergone a radical transformation in recent years that has drastically changed the way different local systems interconnect, interact and exchange information. This transformation has been boosted by the incorporation of new Internet of Things (IoT) technologies that have allowed for the creation of local networks, which are cost-effective and viable from the point of view of energy consumption, as many elements are powered by batteries [1]. However, it comes also with different challenges as it has led to the transition from a model segmented into different categories and services independent from each other, to the current trend of multiservice platforms that not only support multiple protocols but also allow for data intercommunication.

**Figure 1:**
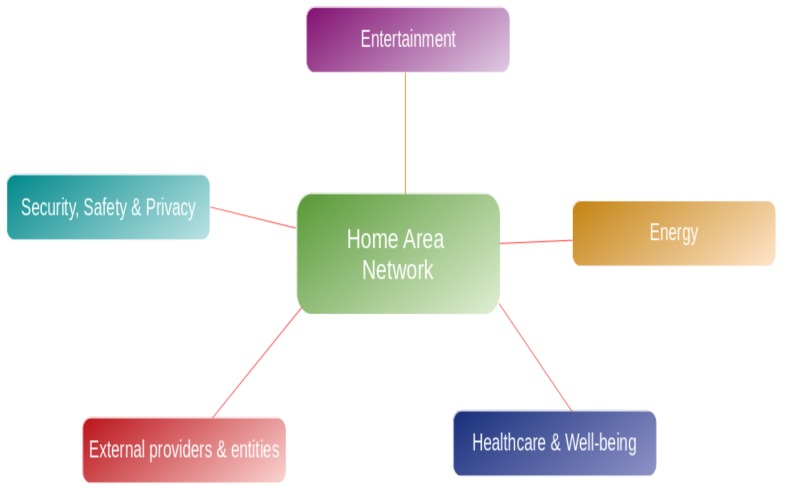
Services offered by smart homes.

This transition has been possible thanks to the support given by the Home Area Network (HAN), which is a type of IP-based Local Area Network (LAN). It connect multiple devices inside a small boundary, and allow the sharing of the resources among them. This concept is gaining popularity due to its main features such as advanced automation, efficient energy management, interoperability between the various devices, particularly constrained IoT devices, and robust security and privacy management. From our perspective, the HAN network inside the smart home should give support to five main categories of services, as shown in Figure 1. However, based on the current status analysis, the author found it promising to elaborate more on two of the smart home services, namely energy and the healthcare. By adopting these services as the study objects, authors would like to investigate possible paths of developing such domains in terms of efficient structures and real demonstrations.

### 1.1. Background

As outlined in Figure 1, the first important service in a smart home is the *Entertainment* domain. It is regarded as the variety of devices located in the smart home that provides comfort, joy and relax. The main types of smart devices on the market that deliver this kind of service are multimedia (such as smart TVs, and connected speakers), hubs and controllers that help in controlling the gadgets and appliances in the house, lighting (e.g., intelligent dimming for energy savings) [2,3] and heating/cooling through smart thermostats that can bring further energy savings [4]. Such services, in particular the multimedia related ones, requires a higher network demand for low-latency, high-capacity, real-time and high Quality of Services (QoS). What is more, they consume most of the network bandwidth and require higher processing and storage resources.

The *Healthcare and wellbeing* is considered as another key feature of the smart homes. Health-monitoring systems in smart environments are gaining increasing attention in order to provide complementary solutions to traditional healthcare services. The advances in wearables, smartphones and medical sensors (blood glucometer, oximeter, blood pressure, electrocardiogram sensor (ECG), wearable sensors, etc.) allowed the possibility of collecting a large amount of real-time health data. Subsequently, these data can be processed in order to provide a comprehensive and predictive picture of an individual wellbeing and health, with the aim of maintaining better health outcomes and conducting early interventions to anticipate health needs.

Another key feature in smart homes is the *Energy Management*. The main goal here is to reduce energy consumption costs and to increase the level of user’s comfort regarding household task scheduling (TSC) and thermal comfort (TC) in the smart home. The main function of the Home Energy Management System (HEMS) is to optimally control the operation of in-home devices and appliances concerning different objectives (e.g., minimizing energy costs and maximizing comfort level). For this aim, it considers the measures provided by smart meters, the information on the operation of the devices, users’ requests and external signals such as energy prices and meteorological information. The HEMS can also use information about the presence of people over a period and infer an occupancy model of the residents. This would enhance management of the system within the scheduling period. Furthermore, the occupants of the smart home are very concerned about their *Security, Privacy and Safety*, which is a very important aspect that needs particular attention. Firstly, since users are always worried about their safety and the one of their family and their belonging, several solutions have been proposed on the market in order to respond to this need, which are mainly: smart surveillance cameras (i.e., Cisco), smart locks (i.e., August), smart trackers (i.e., Tile), smart firewall (i.e., Cujo), etc. Secondly, the user’s data must be protected from any external threats. For this reason, several measures need to be taken into account when building such a network, which are mainly: the *authentication* by verifying the users’ identities, the *confidentiality and Integrity of the data* by encrypting all the exchanges and with the third-parties (i.e., external service providers), and last but not least, the *access control* to allow only the authorized users to access the services of the smart home. In this regards, access delegation should also be considered, as external persons coming to the smart home might need to have access to some basic services inside.

**Figure 2:**
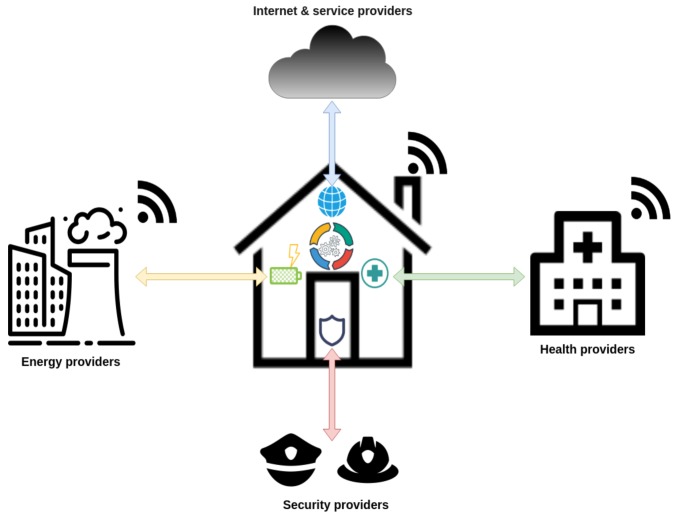
Interactions with external providers.

Last but not least, we argue that the interactions with the *External Provides* needs also particular attention since it comes with its own challenges and issues, eventually the security and privacy ones. These might comprises any service provider for the aforementioned categories, as well as complementary services, as shown in Figure 2.

### 1.2. Contributions of the Paper

Generally, this paper proposes a design together with an implementation of the next generation smart homes, where heterogeneous data coming from multiple sensors (medical, wellbeing, energy, contextual, etc.) and house equipment (smart fridge, smart TV, etc.) are being managed, secured and visualized.

More precisely, and in light of the literature review, this paper aims at proposing a practical structure for integrated management of energy, wellbeing and health in future homes. In this way, it will combine different communication infrastructures such as wired connections, Bluetooth and WiFi. It will also provide a common data representation in order to facilitate the integration of different services in a unique platform. Finally, it will deal with the security, safety and privacy issues due to the sensitive nature of energy and health data. To summarize, our main contributions, that will be detailed later in the next sections, can be listed as follows:

1. A layered smart home structure is proposed where security as a priority is implemented at the application level by encrypting all the traffic flowing between different components. The smart home architecture provides as an initial step two types of services, which are the energy management and the healthcare management services, toward the management of all the other services that can be present in the smart home in an intelligent way.
2. Practical healthcare services are introduced and implemented to allow the occupants of the smart home to first monitor their vital signs for both healthcare and wellbeing purposes and also to provide the possibility of performing remote consultations with a doctor from homes using a simple user interface.
3. An intelligent energy service is proposed to enable monitoring and management of the energy consumption inside the smart home. The main objective is to reduce energy consumption costs and to increase the level of user’s comfort regarding household task scheduling (TSC) and thermal comfort (TC) in the smart home through the HEMS.
4. A simple user interface is implemented in order to visualize all the data exchanged inside the smart home, together with a secure access to the different services.
5. A notification system is designed in order to send alerts to whom concerns (user, family, doctor, etc.) when an abnormality is detected. Additionally, an android application communicating via Bluetooth Low Energy (BLE) is implemented in order to receive such notifications.

This paper is structured as follows: a review of related literature mainly for the healthcare and the energy management in the smart homes is presented in Section 2. Next, the proposed smart home architecture together with the main communication protocols, the structure of the centralized gateway of the smart home and the main components of the proposed security layer, are introduced in Section 3. Section 4 presents the main services incorporated in the smart home, followed by the main algorithms that have been used to test and provide reliability to the previous services. Then, in Section 6 a detailed explanation of the proof of concept is provided. A discussion regarding the main challenges encountered and solved in this paper together with a discussion of the possible research directions is proposed in Section 7. Finally, Section 8 concludes the paper and draws the future works.

## 2. Related Works

As mentioned earlier in this study, different services can be offered in smart home environment. In this section, we aim at elaborating more on two of the smart home services, namely energy and the healthcare and provide a review of recent literature in these subject areas.

### 2.1. Healthcare

In the literature, several works have been done in order to provide remote healthcare services for the patients in their homes as in Ref. [5], where the authors present a cloud-based web service-oriented architecture relying on a ”home system” for the collection of information from a heterogeneous set of devices, and which includes the core elements of the REST service platform and many features required for the healthcare services to operate. However, the drawback of the work is that they do not deal with the data security issues, which is one of the main concerns of the users at any architecture dealing with health related data. In Ref. [6], the authors present their research work in a project called SPHERE (Sensor Platform for Healthcare in a Residential Environment) developed in UK, with the aim of providing an interdisciplinary work to build a generic platform that merges complementary sensor data to generate rich datasets that support the detection and management of various health conditions. In particular, in the case of the Ambient Assisted Living (AAL) and the case of continuous management of health conditions in smart homes. However, the only major drawback of the project, as far as we understood, is that they do not provide a deep and detailed security analysis for the overall system. In particular, there is no analysis regarding the access control in the system, and to the different IoT medical devices. Ref. [7], presents a smart e-health gateway to be deployed for ubiquitous health monitoring systems especially in a clinical environment. The proposed gateway can manage various sets of smart medical objects that can be for instance present in the different rooms of a smart home or a smart hospital. However, the development was focused only on the remote monitoring of the patients. Moreover, neither the privacy aspects nor the data visualization of the heterogeneous sources in a smart home was addressed in that work. Whereas [8] proposes an IoT-based remote health monitoring architecture (IReHMo), with the aim of taking care of the health of elderly people without compromising their convenience and preference of staying at their home. They also propose to solve the issue of the efficient transmission of healthcare data within the limit of the existing network infrastructure, especially in remote areas in Sweden, using CoAP. However, from the security point of view, they only deal with the encryption of the data, and other issues such as authentication and access control, in addition to the privacy, are not mentioned. In Ref. [9] , the authors propose a remote patient health monitoring in smart homes by using the concept of fog computing at the smart gateway. The data are gathered from the IoT sensors through the data acquisition layer, and analyzed at the fog layer. A notification is sent to the cloud layer only if an adverse event is triggered. However, they also do not consider the security and the privacy aspects, even though their architecture deals with sensitive health data. The authors of Ref. [10] presents an emotion-aware connected healthcare system. It captures speech and images of the patient in a smart home environment and then processes them in order to detect the different emotions such as pain. The latter may for instance trigger the intervention of a caregiver.

To summarize, the major drawback of the previous works is obviously the lack of deep analysis of the different security aspects, in particular regarding the access control. In our proposition, the architecture of the smart home considers the security as a priority by implementing a security layer at the application level and by encrypting all the traffic flowing between the different components.

### 2.2. Energy Management

Over the past few years, many research works have been conducted in this subject, which mainly focus on the use of data from different energy resources and scheduled these resources accordingly to optimize the energy consumption. In most of these studies, a standalone Energy management System (EMS) is implemented to collect data from the local resources in order to optimize their operation. As an example, authors in Ref. [11] propose a method for optimal operation management of smart home appliances considering the real-time energy prices. By the same token, a solution is proposed in Ref. [12] to reduce the energy consumption cost and the peak demand of a residential unit through integration of an EMS. The proposed design benefits from wireless communication and smart metering infrastructure in order to make an efficient scheduling for the electric appliances. Another work in Ref. [13] provides a comprehensive review on HEMS, with a particular focus on DR programs, the smart technologies used, and the load scheduling controllers. The problem of optimal residential energy management in both thermal and electrical zones is also tackled in Ref. [14] where the HEMS is designed to minimize the sum of energy cost and thermal discomfort cost in a long-term horizon. The authors of Ref. [15] describe an autonomous energy management-based cost reduction solution for peak load times using a HEMS, by controlling the smart home appliances linked to the smart meter. In the same subject area, Ref. [16] proposes a self-learning home management system (SHMS) using computational and machine learning technologies in order to enhance the system capabilities such as price forecasting, price clustering and power alert system. The overall system is composed of a HEMS, a Demand Side Management (DSM) system, and a supply side management system, inside a real smart home in Singapore. As for the smart metering systems, Ref. [17] presents the integration of the advanced smart metering infrastructure (AMI) in the context of the smart building with an EMS in order to enhance the management capabilities of each individual consumer, using the daily energy profiles collected from this architecture.

As for the application layer, the authors of Ref. [18] propose a centralized system implemented in software application to manage the home appliances through a Wireless Sensor Network (WSN). It uses several management algorithms based on consumption models combining timing schedule, power, temperature or ambient light measurements and prioritization. The proposed system uses stochastic models for the prediction of household consumption and generates dynamically proper power limits depending on the day and the week periods. Also, the research work in Ref. [19] describes the development of an application to control the in-home energy consumption. The proposed application performs the management of energy by means of a wireless personal network based on ZigBee standard and predicts the demand using stochastic models hosted on a Web Server.

Although different aspects of energy management in smart home environments have been studied in the reviewed literature, the major drawbacks of the mentioned works are threefold: (i) most of the designed infrastructures are based on local area networks where interoperability of various devices with different communication technologies cannot be supported, as also discussed in Refs. [20,21,22]. (ii) The majority of the developed architectures cannot interpret and process the sensed and measured information from different nodes at home. They also suffer from lack of data management systems to be run by the HEMS to manage and store contextual data for later retrieval. (iii) None-of the aforementioned research works deal with the security and privacy issues associated with implementation and realization of the energy management systems, where diversified physical sensing information must be stored, processed and dispatched to the actuator components and users.

## 3. Proposed Smart Home Architecture

The smart home vision aims at enabling, to a certain extent and in an independent and easy way, a better interaction with the user, in order to improve the quality of life of the residents. This work represents real experiments carried out in a smart home built at Aalborg University, Denmark.

### 3.1. Architecture Layers

**Figure 3:**
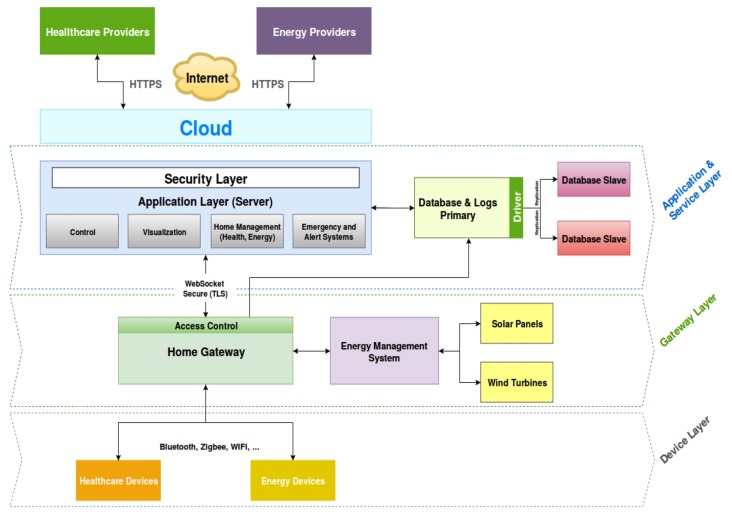
Next generation smart home architecture.

The architecture is a preliminary design toward such vision. The proposed architecture is composed of four layers, as shown in Figure 3.

**The Device Layer**: represented by all the IoT devices in the smart home, which can be sensors, actuators or even connected smart home appliances. In this contribution, a special focus is given to the medical IoT devices and smart plugs in order to measure the energy consumption of the different components. Moreover, renewable energy sources are also equipped with sensors that measure the current, voltage, power and send the data using the ZigBee protocol. These data can be then visualized or used in the EMS at the application layer for different control and forecast strategies. Moreover, the smart meters are used in order to collect the energy, heat, and water consumption inside the smart home, and then to send them to the data concentrator located in the smart home gateway.

**The Gateway Layer**: represented by the smart home gateway. It provides the ability to communicate with the different smart devices in the smart home (energy, health and smart meters), using different communication protocols (e.g., Bluetooth, WIFI and Zigbee). Details about the gateway will be provided in Section 3.3. Different stack of communication protocols were deployed in order to enable the interaction with different kinds of IoT devices. The gateway also interacts with databases in order to store the data coming from all the sensors, errors, alerts and operational logs. From this layer a secure web socket is established between the application server and the home gateway in order to exchange data and commands in a secure way. Both components are located in a private local network of the smart home and connected to a secure WiFi.

**The Application and Service Layer**: provides the main services of the smart home, in particular, the ones related to the health and energy, together with the possibility of interacting with external entities, and controlling different smart home appliances. Through a simple visualization interface, it provides all the data collected from the different sensors of the smart home and the possibility of controlling and managing them. Another important feature is the ability to collect alerts and error notifications and to react accordingly. A security layer is added on top of the application layer so users have to authenticate in order to be authorized to access certain resources and data for each user. What is more, all data exchanges are encrypted using AES128 to provide confidentiality and integrity. A Node.js server providing a Web API is deployed. This server can either extract real-time data directly from the gateway for the case of remote tele-consultation as will be explained later, or query a database for historical records. Thanks to the selected development technologies, all these services can be deployed on the Cloud in a seamless way. Moreover, in order to receive notifications and alerts from some particular sensors (in particular from the most critical ones), a simple android application was developed. The application uses Bluetooth Low Energy (BLE) in order to communicate with the gateway, and can either read, write or subscribe to a particular sensor to receive notifications. For the energy part, the EMS is used in the application layer in order to infer control strategies from the measured data (i.e., sensors and smart meters data collected through the utility) and use the smart plugs to carry them out.

The third-party providers are added in order to connect the smart home with the different entities and service providers in the city. It can be also seen as a tentative to build a smart city network, where each smart home is connected to the different smart facilities of the city. For instance, and as we will explain later, a connection to the healthcare provider can be established to allow for quick interventions in emergency situations, as well as another service for weather forecasting, used by the EMS to estimate the daily consumption profile.

### 3.2. Communication Protocols

In the context of the smart home, every object, device, home equipment, etc., needs to be able to exchange information with multiple devices and to be connected to the local network of the smart home, in order to interact and control them in an efficient way. However, there are currently no common standards and specifications regarding a unified communication protocol for all these devices. The same myriad of protocols can be found for the IoT. Hence, issues such as interoperability, compatibility and security still exist and require more efforts to achieve such goals [23]. Efforts are made by the WoT community [24] in order to abstract all the complexity of the connectivity part of the smart objects, by providing a standard application layer based on Web standards to simplify the creation of IoT applications.

In our experiments, a special focus was given to the most widely used wireless personal area communication protocols such as BLE and Zigbee, as well as WIFI for less constrained devices and Ethernet as a wired protocol. Mainly, a gateway, exposing a REST API and implementing several communication stacks, is used to interact with the different smart objects.

### 3.3. The Smart Gateway

The interoperability issues can be solved, by using a secure gateway, implementing the main communication stacks in order to be able to communicate with all the devices. The gateway also acts as a data aggregator that processes data traffic coming from different devices in the home network, independent of the means of physical transmission, i.e., wired or wireless. Since the gateway is able to communicate either by using a wireless communication protocol (i.e., Zigbee, Bluetooth, etc.) or by using Ethernet directly linked to the devices. The data provided by this gateway will be then sent to the application server.

**Figure 4:**
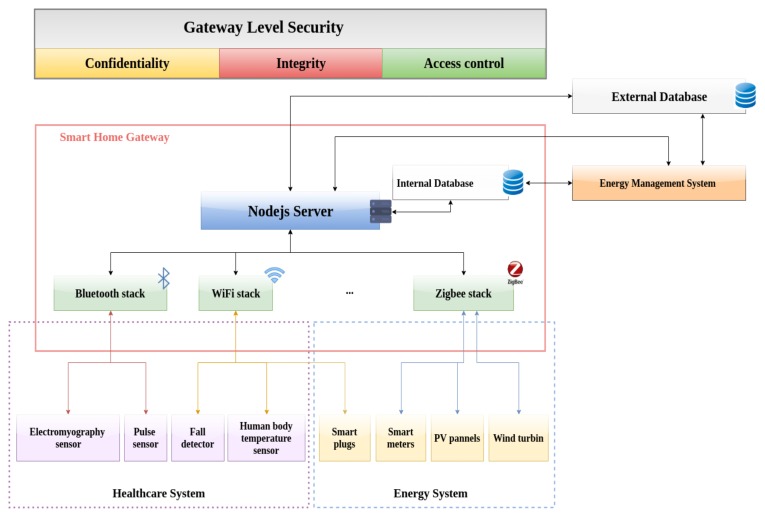
next generation smart home gateway.

As shown in Figure 4, the smart gateway is a Node.js server running inside a raspberry pi 3, for experimental and open source purposes. The raspberry is also connected to other gateways in the smart home in order to have a full view and to manage them all in a centralized way. The gateway provides the possibility to interact with various devices using different communication protocols toward an interoperable architecture. Since the scope of the contribution is narrowed down to the health and energy services, the connected devices can be divided into two categories: the health-related medical devices, such as pulse sensor, muscle sensor, temperature sensor and fall detection sensor using an accelerometer, and the energy-related devices, which can be smart plugs, energy harvesting sensors or smart relays. Moreover, for each sensor, an actuator is attached. The idea is that if an abnormality is detected, a command is sent to the actuator in order to perform some actions. For Proof of Concept (PoC) purposes, “Servo Motors” and “LEDs” are used. The gateway is also connected directly to a database in order to store the data coming from the sensors, the errors and the alerts. A security layer is added to the gateway in order to protect the data against unauthorized access (using an access control) and against eavesdropping and tampering (using encryption).

## 4. Services

### 4.1. Security Layer

**Confidentiality and Integrity**: in all the cases, and in particular in sensitive domains, such as the health, data exchanges between the parties need to be kept confidential so only the authorized ones are able to access them. A falsified or altered message must be detected. The most commune solution is to use strong encryption algorithms and strong security protocols that implement those algorithms. In our PoC, and as recommended in the RFC3565 [25], AES128 encrypts the data exchanged between the gateway and the application server, using pre-shared keys.

**Authentication**: in order to verify the identity of the user’s, for the PoC, an authentication middleware called “passport” [26] is used. Passport is a simple, unobtrusive authentication for Node.js. It provides several authentication strategies, in our architecture a local authentication strategy is used, which is based on user credentials (username, password). However, other strategies can be developed later, in order to provide more robust authentication schema such as using single sign-on using an OAuth provider (i.e., Facebook or Twitter).

**Access control**: traditionally, the access control focuses on the protection of data based on the identity and attributes of the users. Access control is used to protect front-end and back-end data and system resources by adding restrictions on who can access the data, which resource they have access to and what operations are allowed to be performed on the data. Ideally, access control prevents unauthorized users from viewing, modifying or copying the data. For computer network systems, and in order to facilitate access control, standard authorization models are proposed, such as Access Control List (ACL) [27], Subject/object access control matrix [28], Multilevel security using information flow [29], Role-base access control (RBAC) [30] and Attribute-based access control (ABAC) [31], dynamic authorization model have been suggested [32] capability-based systems [33]. In this contribution, RBAC was chosen in order to provide a PoC. ABAC seems more suited for the general smart home use case, however, further studies need to be done in order to determine the best model. The access control is done in the gateway, after a successful authentication of the user, the application server requests the gateway if the user is authorized to access the resources or not. If yes, the user will be redirected to the web page containing all the data collected from the gateway. Otherwise, an error will be raised.

### 4.2. Healthcare Services

For the health services, the idea is to be able to provide remote health services for the patients in their homes, through several use cases, such as:

**Wellness follow-up**: the patients can use the medical devices in their home in order to follow their health status for wellness purposes through the visualization interface. For instance to check the body temperature, or to regularly check the cardiac parameters such as the Heart Rate.

**Health issues self-follow-up**: where the patient with chronic disease, such as diabetic, can follow its health status daily using its medical sensors. In case of detection of an abnormality, two cases are presented here: For independent patients, they can call the medical service (a doctor for instance). If the patient is dependent, in this case, either simple solutions for calling the medical service such as a button can be used, or an automatic abnormality detection algorithm can notify the medical service.

**Remote medical consultation or Tele-consultation**: patients can consult their doctors remotely, using multimedia enhanced with IoT contextual health data [34]. This can be a solution to solve rural areas issues regarding the access to medical services. This solution can also solve the problem of medical follow for elderly and persons with chronic diseases in rural areas, where usually they do not need to be physically present in front of the doctor in order to have the follow-up. Hence, reduce the stress, tiredness, time and money for them.

**Remote monitoring**: where the patient is usually equipped with medical connected devices, and in case of detection of an abnormality in the behavior or the vital signs of the monitored persons, an alert is sent to the closest medical service [34].

Additionally, one of the main issues is to be able to notify the concerned parties in case of detection of an abnormality in the behavior of the user (e.g., fall detected) or from the collected data. In our PoC, all the alerts and errors are sent to a central database, which can be for instance the database of the hospital. Moreover, solutions such as sending emails and SMSs are feasible and can be included in future work.

### 4.3. Energy Management Services

The proposed HEMS introduces a hybrid AC/DC platform with different means of local generation (such as wind turbine (WT) and roof-top PV panels) and energy storage as well as controllable loads, here named as IoT devices (e.g., smart washing machine, tumble dryer, freezer, etc.). Moreover, the platform provides practical, optimized and comfort-aware energy management solutions for residents using real-time control and monitoring systems.

## 5. Services Testing and Reliability

The previously mentioned services are then tested through a set of algorithms in order to validate our approach, for instance in order to detect new alerts (e.g., fall detection). Moreover, since we argue that the proposed services must be reliable, a reliability discussion, mainly regarding the databases and the different sensors is provided as a first step. Future works may provide a deeper discussion regarding these two aspects.

### 5.1. Algorithms

To validate our approach, we had to test several use cases inside the home. We developed two elementary algorithms (which can be improved in future work) related to the e-Health (fall detection and temperature evaluation).

#### 5.1.1. Fall Detection

Several proposals and studies have used accelerometers to objectively monitor a range of human movement [35,36], for instance, to measure metabolic energy expenditure, physical activity levels, balance and postural sway, gait, and also to detect falls. These systems have usually a hardware part, which is attachable to the body of the patient (usually around the waist), and a microcontroller, such as Arduino in our case, which is used to classify the person’s actions and to detect any possible falls.

**Figure 5:**
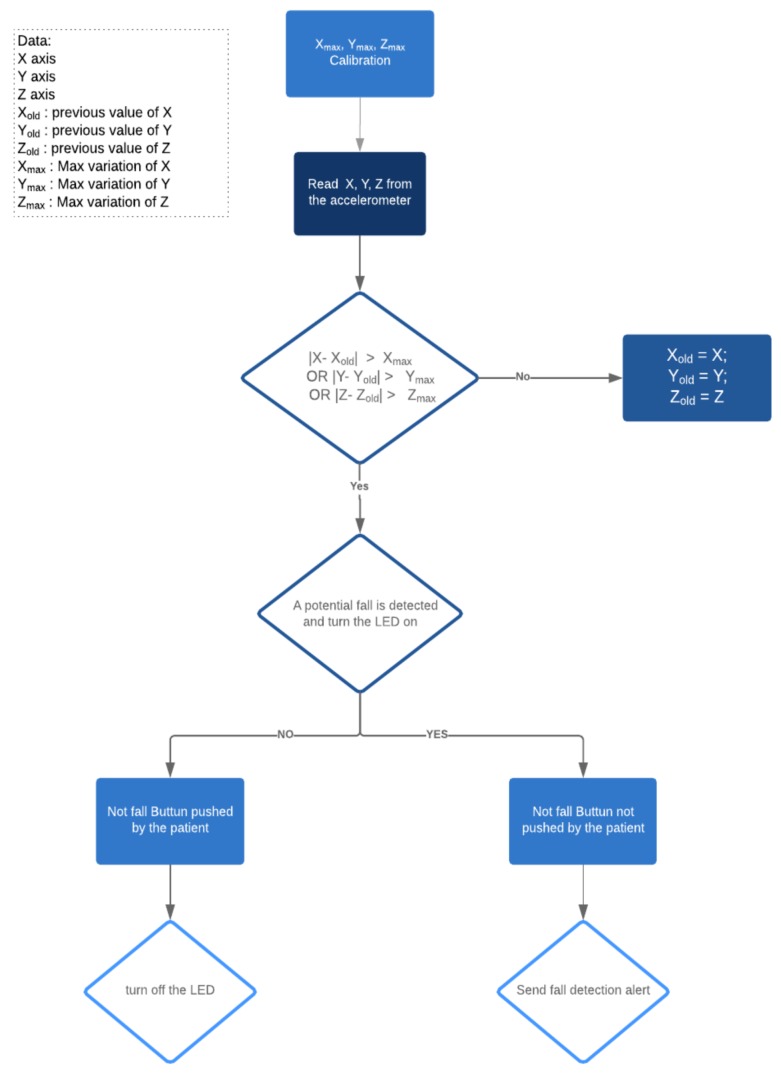
Simple fall detection algorithm using an accelerometer.

In our demonstration, a three-axis accelerometer ADXL330 board is used. Additionally, a simple algorithm, represented as a flowchart in Figure 5, is used in order to detect quick variation of one of the values of the accelerometer (*X* or *Y* or *Z* axis), and upon exceeding of a certain limit, an alert is generated and sent from the controller to the application. A LED is lit when a fall is detected. Additionally, a button also can be used in order to prevent fault positives, where the user is given 30 s to push the button in case of normal movement. A calibration function is used in order to detect the maximum values of each axis of the accelerometer. The value in this case are collected every 1 millisecond, as long as the calibration button is pushed, and usually it takes up to 1 s to get the maximum values for each axis. Generally, the accelerometer values are collected each 2 s.

#### 5.1.2. Wellbeing Monitoring Sensors

For the data collected from these two medical devices, an evaluation is done in order to decide whether the measured data is normal or not. However, a classification of the data needs to be done to contextualize the information. For the pulse sensor, depending on the age and the activity of the user, measured data can have different meanings in each case. For instance, for a person aged more than 12 years, for a normal activity, the normal heart rate is between 60 and 100 bpm (beat per minute) [37]. As for the thermometer, for babies and children, the average body temperature ranges from 97.9 °F (36.6 °C) to 99 °F (37.2 °C), for adults the average body temperature ranges from 97 °F (36.1 °C) to 99 °F (37.2 °C), and for adults over age 65, the average body temperature is lower than 98.6 °F (36.2 °C). Hence for instance a temperature higher than 100 °F (37.8 °C) (mouth readings) is a sign of a fever.

For our demonstration, a value which is considered as abnormal triggers an alert which will be sent to the application layer and visualized to the user. In this case, the concerned age segment was for adults, and a temperature above 40 °C is considered abnormal. Moreover, the idea is to send this alert to the related health provider either through a notification. SMS can be also sent to the relative of the person in particular if the concerned person is a dependent one.

#### 5.1.3. Energy Monitoring and Control

The operational pipeline in this smart home platform is the EMS that controls the operation of in-home devices and appliances optimally with regards to different objectives considering the information about task operating status, user’s requests and network signals received through the AMI system. The AMI by itself includes two layers namely physical and utility layers. The physical layer includes the smart meters and the data concentrator that collects the measurements periodically and provides a dataset of data with synchronized timestamps using a Network Time Protocol (NTP) server. The communication between the smart meters and the data concentrator is based on the standard EN 13757-5 that implements a radio mesh topology. The second layer in AMI topology integrates the logical software provided by the Kamstrup OMNIA suite, to ensure efficient interoperability with the AMI network. This software communicates over Ethernet with the data concentrator and implements all the back-end processes that are necessary to configure the system, perform on-demand operation readings and capture the periodic records.

To keep a meaningful balance between the energy saving ($Obj_{1}$) and a comfortable lifestyle ($Obj_{2}$), EMS should solve a multi-criteria decision-making problem as [38]:

(1) $$\begin{array}{c} Obj_{1}\left\{Min.EnCost=f\left(\rho_{el}^{t},\rho_{gas},P_{grid}^{t},P_{DG_{i}}^{t}\right)\right\}\forall t\in T,i\in N_{DG} \end{array}$$

where EnCost denotes the energy consumption cost of the smart home which is a function of power exchanged between the building and the utility at time *t* ($P_{grid}^{t}$), power produced by domestic generation unit *i* ($P_{DG_{i}}^{t}$), and the real-time grid electricity and natural gas prices, respectively ($\rho_{el}^{t}$ and $\rho_{gas}$). Moreover, the second objective which is formed based on the user’s convenience level about household task scheduling ($C_{task}$) and thermal comfort level ($C_{thermal}$) can be modeled as follows:

(2) $$\begin{array}{c} Obj_{2}\left\{Max.Comf=f\left(C_{task=},C_{theraml}\right)\right\} \end{array}$$

(3) $$\begin{array}{c} C_{task}=\Sigma_{t\in T}\Sigma_{j\in M}\left(\omega \left(j\right)SL\left(t,j\right)\right) \end{array}$$

(4) $$\begin{array}{c} C_{thermal}=\Sigma_{t\in T}CL\left(T_{in}\left(t\right)\right) \end{array}$$

where, SL(t,j) is the user’s satisfaction level when task *j* is executed at time *t* and CL(t) is the level of thermal comfort (regarding the indoor temperature $T_{in}$) observed by the inhabitants at each time step. More detailed information in this regard can be found at [39,40].

### 5.2. Reliability

**Figure 6:**
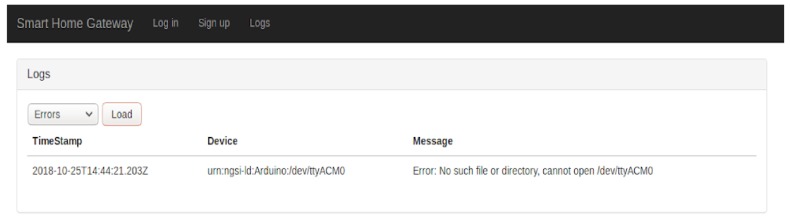
Undetected sensor in the log.

**Undetected Sensor**: the system should detect when a sensor is not connected. In this case, an error is raised and sent to the application, as shown in Figure 6. An unavailable sensor, for certain reasons, can have serious consequences, where for instance in case of home monitoring of a patient, if the fall detection sensor is not available, the fall cannot be detected, or in case of a smart plug which is not working, wrong energy consumption values will provide. In the current state of the work, our system detects if a sensor is missing and send an alert. This alert is displayed on the user visualization interface and registered in the database.

**Figure 7:**
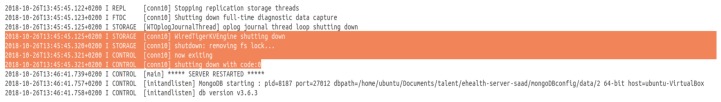
Primary database log.

**Figure 8:**
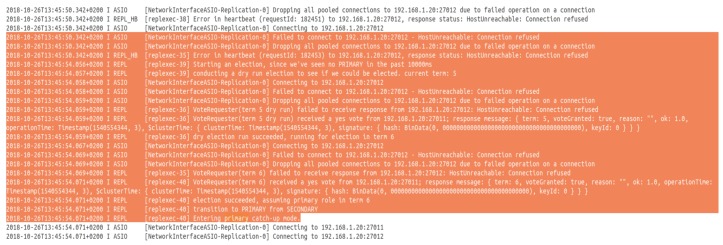
Secondary database log.

**Database fault tolerance**: the architecture implements the NoSQL database MongoDB. To provide more reliability to the system in particular regarding the data provided by the different sensors of the smart home, we used what is called “replica set” in MongoDB. As defined in Ref. [41], “A replica set in MongoDB is a group of mongod processes that maintain the same data set. Replica sets provide redundancy and high availability, and are the basis for all production deployments”. In practice, replication provides redundancy and increases data availability, and to be able to recover in case of a disaster. By creating multiple copies of data on different database servers, replication provides a level of fault tolerance against the loss of a single database server, in addition to better performances, increasing data locality and availability in particular for distributed services. The implantation used the master-slave (primary-secondary) replication, as shown in Figure 3, where the primary server receives all the writes and reads requests, and then replicate the data to the slaves. In the same way, if a primary node is down a reelection is triggered between the members of the replica and if possible, a new primary is selected. The Figure 7 and Figure 8 show this behavior of the primary database and the secondary one respectively when the primary server is down.

## 6. Implementation Results

### 6.1. Hardware Implementation

**Figure 9:**
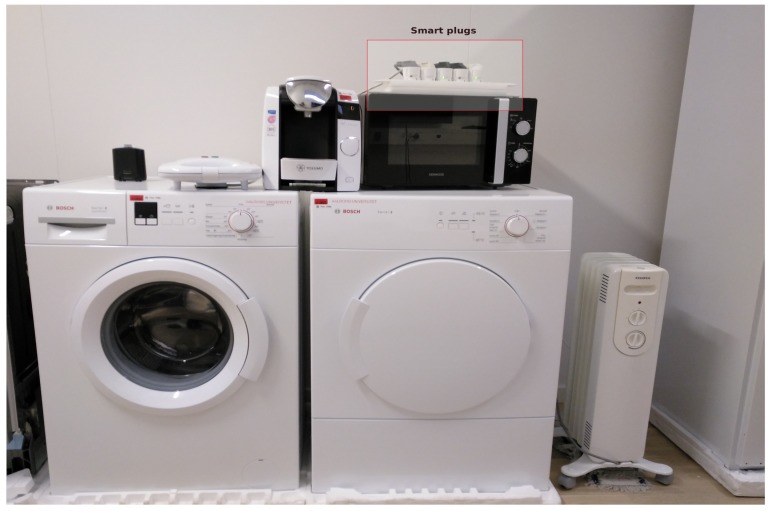
Smart home appliances attached to smart plugs.

As far as the energy devices are concerned, the smart home is equipped with several home appliances as shown in Figure 9. Their energy consumption is monitored using smart plugs from the Danish company Develco. These smart plugs use ZigBee as the communication protocol and are connected to the energy home gateway.

**Figure 10:**
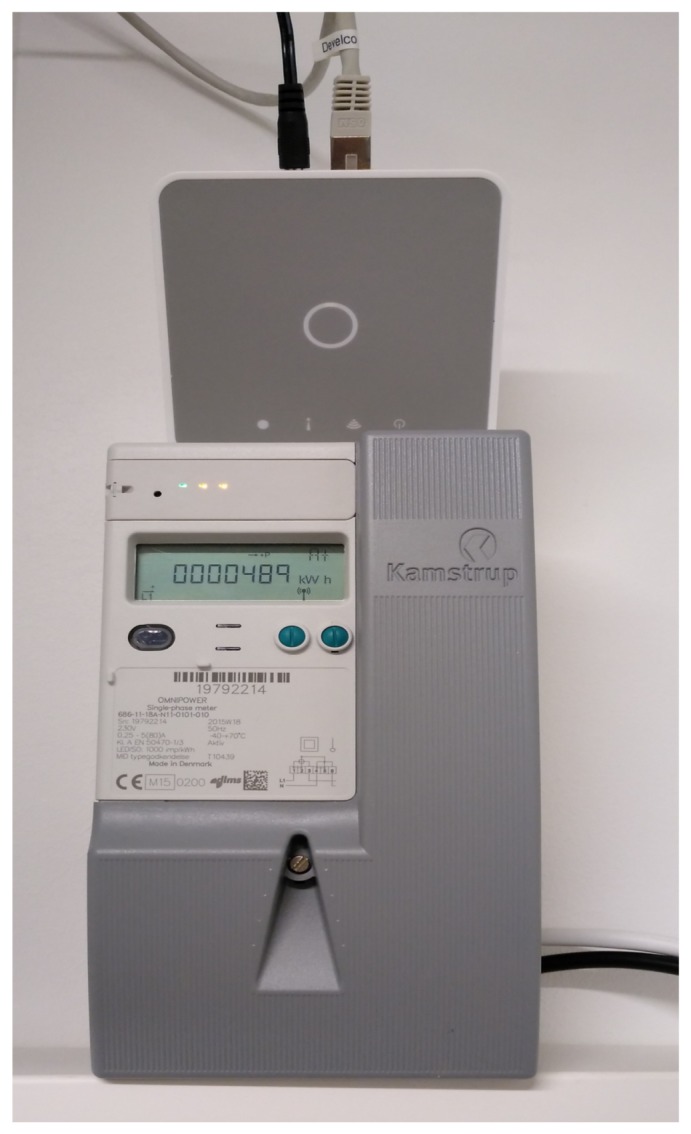
Kamstrup Smart meter.

In addition to the smart plugs, a smart meter from the Danish manufacture Kamstrup was also deployed for measuring the total consumption. This model is widely installed in the Danish homes. The meter is connected to the energy home gateway using a ZigBee module for local measures as well as to a data concentrator, which provides the utility with the demand profiles for billing purposes. The energy home gateway, shown above the smart meter on Figure 10, was also manufactured by the Danish company Develco and contains the embedded ZigBee coordinator attached to an ARM9 CPU which runs a Linux OS. The system is fully flexible and programmable so a JAVA application was deployed. This application exposes a RESTFul API that allows for the data acquisition and actuation on the smart plugs.

**Figure 11:**
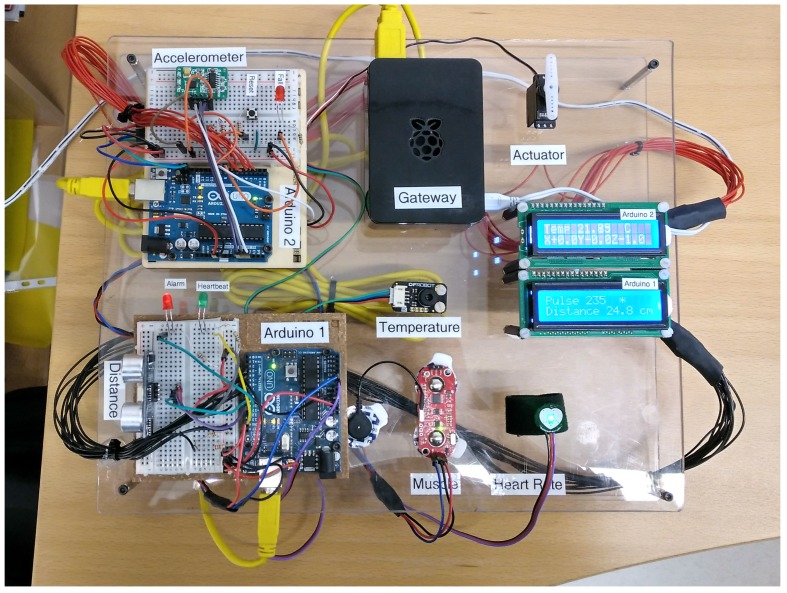
eHealth platform hardware.

On the wellbeing part, a PoC e-Health platform was developed as shown in Figure 11. This system can be used by the patient on a daily basis to monitor his/her health state. It contains several sensors in order to measure the vital signs of the patient. These sensors are connected to two Arduinos UNO. The Arduino 1 measures the heart rate of the patient and the muscle activity. It also includes an ultrasound sensor that detects the presence of the patient next to the platform. The system shows the heartbeat frequency using the green led while the red one shows an abnormality on the patient pulse. The Arduino 2 is responsible for measuring the body temperature and the acceleration in order to detect a fall. The system is connected to an actuator that simulates an alarm based on an abnormal body temperature. It also includes a red led that indicates a fall alarm, which can be reset by means of the provided button. Moreover, two LCD displays are included to provide the user with a local interface.

All of these sensors are linked to the smart home gateway represented by the raspberry pi. This gateway gathers the data from the two arduinos using the USB ports. In addition, it also implements the communication with the energy home gateway.

### 6.2. Data Visualization

**Figure 12:**
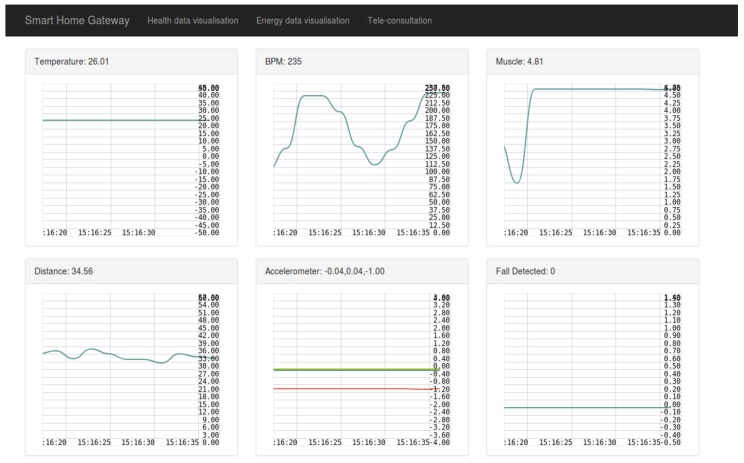
Health data visualization.

**Figure 13:**
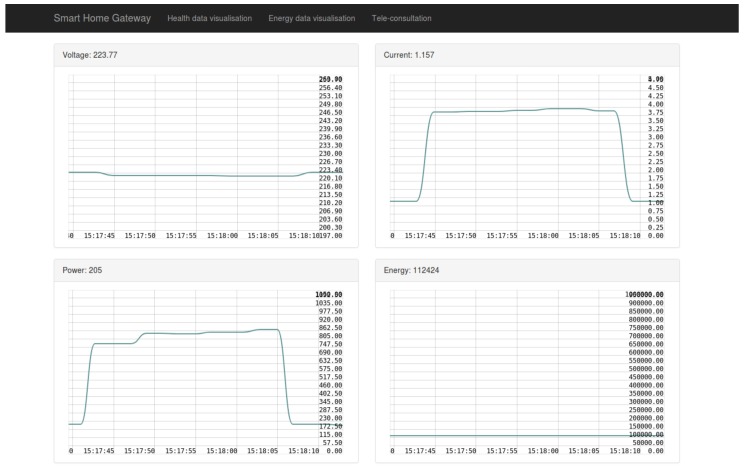
Energy data visualization.

The application layer provides a simple visualization web page to the user with simplified data of each sensor used inside the smart home, and with graphs representation of these data. Since the smart home gateway collects various types of data, it was judged better to separate the visualization of each type in a different page. For instance the health data are displayed in the “Health data visualization” tab, as shown in Figure 12, and the energy data are displayed in the “Energy data visualization” tab, as shown in Figure 13. Moreover, the “Tele-consultation” tab allows the establishment of multimedia session with a remote peer (e.g., a doctor).

**Figure 14:**
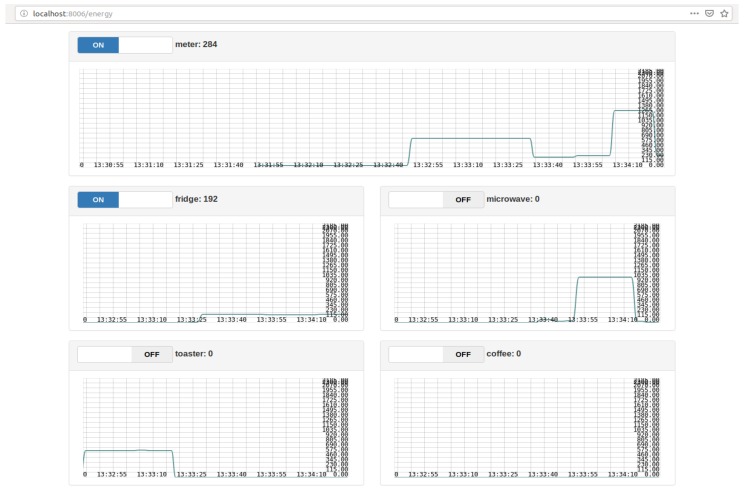
Energy consumption from the smart plugs.

Another portal is added in order to visualize the energy consumption of the different smart home appliances, measured by the smart plugs, all along the day as shown in Figure 14.

**Figure 15:**
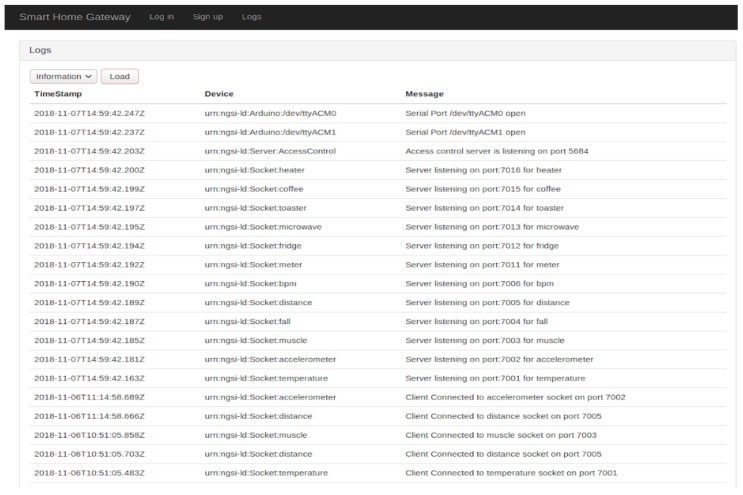
Log page.

Additionally, a login/sign-up portal and other dedicated pages are provided especially for the logs (i.e., Figure 15), errors and the alerts detected (i.e., in case of an abnormality) inside the smart home.

### 6.3. Tele-Consultation Implementation

In our demonstration, a special focus is given to the tele-consultation service. Providing the ability to have remote consultation with a remote medical staff, such as a doctor, is one of the health services that can revolutionize the next generation smart homes, equipped with medical devices. These medical devices can be then used to measure the health status of the patient and to provide insight to the remote doctor in order to provide a better diagnosis for the patient. As explained in detail in Ref. [34], a proposition is to use WebRTC-based architecture enhanced with contextual information of the medical IoT sensors.

**Figure 16:**
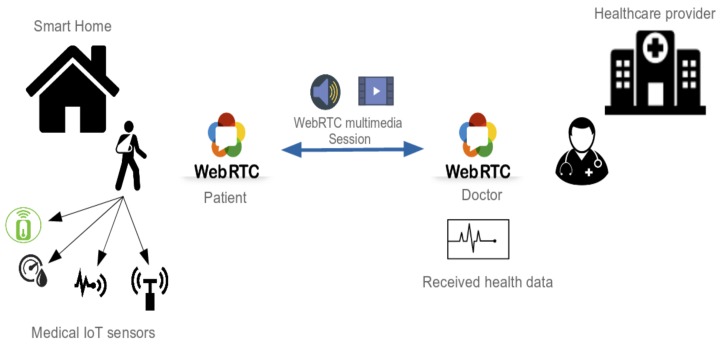
Remote medical consultation in the smart home.

As shown in Figure 16, the patient starts a WebRTC [42] multimedia session with the remote doctor, and in the same time, the wearable medical devices attached to the body of the patient, and also connected to the platform, starts sending real-time health data. Thus, using the information from the direct interaction with the patient and also the information provided by the medical sensors, we believe that the doctor can provide a better remote diagnosis of the state of health of the patient, and thus better remote medical services.

### 6.4. HEMS Implementation

**Figure 17:**
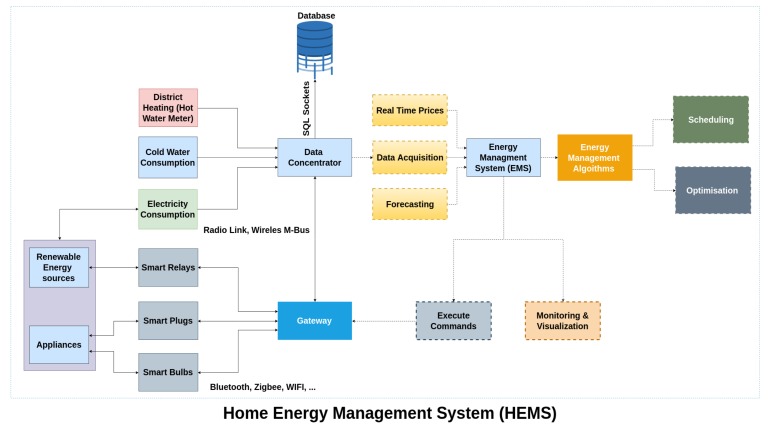
Architecture of the HEMS.

In this section, the architecture of an intelligent EMS is described for a smart home application. As shown in Figure 17, the proposed home energy management system (HEMS) introduces a hybrid AC/DC platform with different means of local generation (such as wind turbine (WT) and roof-top PV panels) and energy storage as well as controllable loads, here named as IoT devices (e.g., smart washing machine, tumble dryer, freezer, etc.).

The physical layer in this platform includes smart plugs and activity sensors such as motion sensor, as well as smart meters. These sensors and devices use different communication protocols including Wi-Fi, Bluetooth and Zigbee to communicate their data to a Gateway. In parallel, the smart meters establish a radio mesh communication with data concentrator to provide the energy measures.

In the data link layer, two parallel tasks are carried out simultaneously. On one side, the gateway collects all the information of the smart plugs and sensors in the MongoDB database. It also provides the necessary interfaces for the actuation commands. On the smart meters side, the data concentrator establishes an encrypted communication with the metering head-end which in this case was tested locally but that usually belongs to the utility company. This head-end system is part of the Kamstrup Omnia Suite and provides a short-term and a long-term storage for the energy information. Another MongoDB database is used to store the on-demand measures as well as auto-collection data for a period up to 3 days. Behind this, a Microsoft SQL database records all the historical measurements. Nevertheless, these measures are always accessed by means of an API provided by the manufacturer.

The application layer is mainly composed by the service to access the HEMS and perform the control strategies. The HEMS is conceived as an external service the user can subscribe to. This system has to be provided it with the necessary input data and in return it will response with the necessary control actions according to the algorithms presented in Section 5.1.

Furthermore, there exist are two main application, which use the data-driven services of a smart home. The first one, which is a LabVIEW-based application, is used for querying on-demand readings from the Advanced metering infrastructure (AMI) network, logging system’s alarms, events or malfunctions. This service could also be used to provide a simple and easy-to-use user interface, with all the significant data. The second application which is the control unit collects all the required information from the energy server and sends optimal reference signals and set-points to related actuators and device-level controllers. This application also provides a supervisory control over the system performance and changes the plan of actions in different working conditions (e.g., normal or faulty) based on a predefined scheme.

The HEMS was developed as a JAVA EE application also interfaced with GAMS solvers in order to carry out the optimization algorithms. To complete a user optimization request, the HEMS must be connected to three main information pipelines from two external services:

- Forecasting pipeline composed of two sub-modules:
  - Weather forecasting pipeline, which use meteorological information in the household and external service to estimate the Wind Turbine (WT) and Photovoltaic (PV) production.
  - Load forecasting pipeline. Based on the historical information recorded by the Smart Meters as well as stochastic models, it estimates the behavior of the consumers in terms of demand for evaluation the most beneficial control actions to be taken.
- Electricity price pipeline, where the utility provides real-time prices for the energy that are included in the optimization process.

It will provide the current information regarding consumption and load state when requesting the HEMS an optimum scheduling. Therefore, after the computation the HEMS API will provide a response with the control action that will be transmitted to the gateway and finally be carried out in each device.

The aforementioned HEMS architecture implemented in the smart home is presented in Figure 17.

## 7. Challenges and Discussions

From our perspectives, the next generation smart home allows the possibility of collecting different types of information, including the contextual data from the immediate environment of the user, from the different sensors and actuators present in the smart home, in order to provide better services to their occupants. In this work, we focused mainly on the possibility of providing two types of services: the wellbeing and e-Health services and the energy services. Each of these two services has different requirements in term of interoperability, deployment, data format, collection and visualization and last but not least security and privacy. As discussed in the different sections, our proposed solution deals with these different challenges, however future works and further investigations needs to be done.

First, the proposed architecture adopts a WoT paradigm in order to interact with the different heterogeneous sensors and actuators in the smart home, by implementing different communication stacks such as Bluetooth, WiFi and Zigbee, in order to guarantee the interoperability inside the smart home.

Then, the designed architecture aimed at facilitating the deployment of the two services, eventually in future works all the other services, by having a control and management layer on top of centralized home gateway that aggregates all the home data and interacts with the heterogeneous IoT devices. The collected data will be then exploited at the management and control layer by the two services. However, a remaining challenge is related to the propitiatory sensors that uses propitiatory gateways, which makes it hard to achieve a centralized control over all the sensors inside the smart home.

An important issue that we encountered during this work is the data format, in particular since the selected services are part of different domains, each of has different requirements and standards. Currently, this still a persistent issue, since there is no standard format for the different type of data in the smart home. A possible solution that we propose is by using a JSON (JavaScript Object Notation) format, which is rich and widely used lightweight data-interchange format. The main advantage of this format is that it is easy for humans to read and write and it is easy for machines to parse and generate. Hence, in our implementation, a data collected from a sensor contains mainly: the urn (Uniform Resource Names), the type of the device, the sensor name, the measured value, the timestamp, and finally the unique ID of the data. These data are then sent both the application server and to the databases. Depending on the targeted use, the data can be either directly retrieved from the sensors, in particular in case of remote medical consultation which require real-time collection of the data, or from the database. Finally these data are visualized using a user friendly interface. Further research needs to be done in order to standardize the most suitable data format for the smart homes.

Last but not least, one of the most pressing concerns for the smart home technologies is regarding the security and the privacy of the collected, stored and transmitted data. In our case, the data may contain sensitive, protected or confidential information, such as the health data, that may endanger residents’ privacy and safety, if breached. Therefore, ensuring strong data encryption, database security, secured communication channels, and so on and so forth is required for the next generation smart homes. In Ref. [43], the authors investigate these issues, by providing a holistic approach of security together with recommendations and good practices for all the stakeholders involved in the smart home environment. The study focuses mainly on the IoT devices inside the smart home (which can be either constrained or with high capability), the interaction and data exchange with remote services and finally the interaction and data exchange with mobile applications. Even though this work implements a security layer in order to guarantee the fundamental security requirement, which are: the confidentiality and integrity, the authentication, and the access control. However, still, he security and the privacy are not the core of this proposition, since, the main goal of this work is the proposition of a new smart home architecture encompassing both the energy and the health services. Therefore, a deeper security and privacy analysis is subject of future work.

## 8. Conclusions and Future Works

In this contribution, the architecture of a next-generation smart home was presented, which we consider as a smart space. It encompassed different types of services in order to provide a better quality of life for the occupants, named healthcare and energy management. The two services were implemented, deployed and tested at a laboratory scale environment, but with real-world appliances and systems. Moreover, a user-friendly interface was provided so the user could have a clear visualization of the different data (i.e., health and energy) collected from all over the smart home, together with alert systems in case of detection of an abnormality (i.e., a fall detection). One of the main objectives of such architecture is to link the smart home to the external service providers, such as the energy utility, in order to first improve these services (i.e., to have a cost efficient-energy consumption) and to secondly take a step toward the vision of the smart city. From this work, several perspectives and challenges were identified in order to achieve the vision of the next generation smart home, where the occupant can benefice from advanced, secure, privacy respectful and easy to use services.

A first interesting perspective, related to the previously mentioned healthcare services, is to investigate in such environment is the measuring of the total energy expenditure (TEE). It provides an objective index to track the motion profile of individuals and allows comparing the level of physical activity with the recommendations provided by the healthcare authorities. Wearable technology has attained considerable development are now available. Particularly, a combination of accelerometers and heart rate monitors has been shown to provide a fairly accurate estimation of energy expenditure.

Furthermore, since, one of the objectives of this work was to test the possibility of having multiple services in the smart home, a second perspective is to investigate the challenges of integrating the other services of the other HAN categories such as the management of the security and safety and the management of the entertainment devices (e.g., by using AI for instance) in the smart home and the management. Obviously, each service has different requirements that need to be identified and guaranteed and different standards that need to be followed.

On the other side, connected appliances and devices inside the smart home will produce a significant amount of data that need to be collected, classified, processed, stored, secured and so on and so forth. Solutions including big data [44,45,46] and fog computing [9] can be considered in future works in order to solve some of these challenges. Where the main idea is to take advantage of the computation capabilities of the different devices and gateways that are present in the smart home in order to process the data.

Last but not least, as mentioned earlier managing the security and the privacy in the next generation smart home needs a particular attention [47,48]. Obviously, each service has different security and privacy requirements that need to be guaranteed. In our case, the health sensitive data need more strict security and privacy measures compared to the energy data. Moreover, in the literature there are several isolated works that tries to deal with the security and the privacy issues particularly for the smart homes such as in Refs. [49,50,51]. However, obviously there is a need for a standardization entity that provides dedicated standards regarding the security and privacy in the smart home.
